# Strategies and Future Opportunities for the Prevention, Diagnosis, and Management of Cow Milk Allergy

**DOI:** 10.3389/fimmu.2021.608372

**Published:** 2021-06-10

**Authors:** Benjamin Zepeda-Ortega, Anne Goh, Paraskevi Xepapadaki, Aline Sprikkelman, Nicolaos Nicolaou, Rosa Elena Huerta Hernandez, Amir Hamzah Abdul Latiff, Miu Ting Yat, Mohamed Diab, Bakr Al Hussaini, Budi Setiabudiawan, Urszula Kudla, R. J. Joost van Neerven, Leilani Muhardi, John O. Warner

**Affiliations:** ^1^ Pediatric Allergist Private Practice, Angeles Lomas Hospital Huixquilucan Mexican State, Mexico City, Mexico; ^2^ Department of Paediatrics, KK Women’s and Children’s Hospital, Singapore, Singapore; ^3^ Allergy Department, 2^nd^ Pediatric Clinic, National and Kapodistrian University of Athens, Athens, Greece; ^4^ Department of Pediatric Pulmonology and Pediatric Allergology, Beatrix Children’s Hospital, University Medical Center Groningen, University of Groningen, Groningen, Netherlands; ^5^ Medical School, University of Nicosia, Nicosia, Cyprus; ^6^ Pediatric Allergy Clinic, Pachuca, Mexico; ^7^ Allergy & Immunology Center, Pantai Hospital Kuala Lumpur, Kuala Lumpur, Malaysia; ^8^ Department of Paediatrics, Queen Elizabeth Hospital, Hong Kong, China; ^9^ Pediatric Department Faculty of Medicine, Children Hospital Cairo University, Cairo, Egypt; ^10^ Department of Pediatrics, Abdul Aziz University Hospital, Jeddah, Saudi Arabia; ^11^ Department of Child Health, Faculty of Medicine, Univesitas Padjadjaran, Bandung, Indonesia; ^12^ Department of Pediatrics, Dr. Hasan Sadikin General Hospital, Bandung, Indonesia; ^13^ R&D, FrieslandCampina, Amersfoort, Netherlands; ^14^ Wageningen University & Research, Wageningen, Netherlands; ^15^ Medical Affairs, Friesland Campina AMEA, Singapore, Singapore; ^16^ Inflammation Repair and Development, National Heart and Lung Institute Imperial College, London, United Kingdom; ^17^ Paediatrics, University of Cape Town, Cape Town, South Africa

**Keywords:** partially hydrolysate, intermediate extensive hydrolysate, extensive hydrolysate, prevention, management, cow milk allergy, hydrolysate step-wise approach, early oral tolerance development milk ladder

## Abstract

The prevalence of food allergy has increased over the last 20-30 years, including cow milk allergy (CMA) which is one of the most common causes of infant food allergy. International allergy experts met in 2019 to discuss broad topics in allergy prevention and management of CMA including current challenges and future opportunities. The highlights of the meeting combined with recently published developments are presented here. Primary prevention of CMA should start from pre-pregnancy with a focus on a healthy lifestyle and food diversity to ensure adequate transfer of inhibitory IgG- allergen immune complexes across the placenta especially in mothers with a history of allergic diseases and planned c-section delivery. For non-breastfed infants, there is controversy about the preventive role of partially hydrolyzed formulae (pHF) despite some evidence of health economic benefits among those with a family history of allergy. Clinical management of CMA consists of secondary prevention with a focus on the development of early oral tolerance. The use of extensive Hydrolysate Formulae (eHF) is the nutrition of choice for the majority of non-breastfed infants with CMA; potentially with pre-, probiotics and LCPUFA to support early oral tolerance induction. Future opportunities are, among others, pre- and probiotics supplementation for mothers and high-risk infants for the primary prevention of CMA. A controlled prospective study implementing a step-down milk formulae ladder with various degrees of hydrolysate is proposed for food challenges and early development of oral tolerance. This provides a more precise gradation of milk protein exposure than those currently recommended.

## Introduction

Food allergy constitutes the second phase of increases in the prevalence of allergic diseases that have occurred over the last 60 years. Initially, asthma and allergic rhinitis increased mostly in affluent countries during the second half of the 20^th^ century. In some countries, the prevalence of these respiratory allergic diseases have plateaued or are falling such as in Korea, Morocco, Thailand, Australia and Brazil (1, 2). However, the prevalence of food allergy has increased over the last 20-30 years (3). Food allergy affects 6-8% of children worldwide and the disease burden is highest for infants and preschool-aged children (4). Cow’s milk is one of the most common and early causes of infant food allergy, affecting 1.4-3.8% of young children (5–7). The Europrevall study which used rigorous ascertainment including double-blind placebo-controlled food challenge (DBPCFC) in 12,000 infants aged 24-30 months showed an overall prevalence of 0.59%, ranging from 0-1.3% in different countries (3, 8), while similar rates have been shown in studies from Israel, Denmark and USA (9).

Cow’s milk protein allergy (CMA) can be considered as an umbrella term for different diseases with distinct symptoms, pathophysiology and treatment and can be further classified as immunoglobulin E (IgE)-mediated food allergy, non-IgE mediated food allergy, or mixed IgE and non-IgE mediated food allergy (10). These immunologically mediated allergic reactions to cow milk must be distinguished from milk intolerance where a non-immune mechanism is involved, such as lactose intolerance, and from milk aversion or psychological intolerance. Food intolerance such as lactose intolerance does not involve the immune system but a deficiency of an enzyme to digest lactose. Thus, the diagnosis of milk or lactose tolerance does not involve any immune parameters, while immune-related disease involves relevant immune markers (11).

Accurate diagnosis of CMA is not easy; a detailed anamnesis and clinical history are critical to establishing the diagnosis as often, parents are not aware of various allergic manifestations (12, 13). The gastrointestinal (GI) system is most commonly affected. Some important GI symptoms include: dysphagia which presents with screaming, back-arching, gagging or choking during feeds (may be due to milk-induced eosinophilic esophagitis), vomiting, reflux, food impaction, delayed gastric emptying, abdominal pain, diarrhea, bloody stool, constipation, perianal rash, anorexia, early satiety and hematochezia, in neonates and infants (14). Moreover, discrimination of pathological from ‘‘benign’’ GI symptoms such as vomiting from reflux is often difficult to distinguish.

The Enquiring About Tolerance (EAT) study collected data on parental reporting of non-IgE mediated symptoms such as colic, vomiting, diarrhea and constipation. They found that infants who were encouraged to introduce cow milk and other food allergens from 4 months of age, reported significantly more non-IgE type symptoms such as eczema flares and colic, than the group which introduced allergens to complementary feeding after 6 months of exclusive breast-feeding (8.6% compared with 3.8%, respectively (p <0.001). This could also be due to the presence of confounding factors at such a young age (15).

Exclusively breastfed children are also at risk for CMA. While the continuation of breastfeeding with maternal elimination of cow’s milk protein (CMP) was recommended (13), this has been challenged in a recent publication (16). However, exclusion depends on the child’s symptom and an elimination/challenge protocol should still be done to ascertain CMA (13). For children below the age of 2 years and non-breastfed children, the main treatment in CMA remains avoidance of CMP and the use of extensively hydrolyzed formulae (eHF). The use of other alternative formulae such as soy or rice formulae needs to be considered as well, while amino acid formulae (AAF) is only recommended when the child still has symptoms of CMA on eHF or severe reactions such as anaphylactic shock (17, 18).

As there is a significant impact of food allergies on the quality of life of children and their families including emotional, psychological and financial burden (19–21), further study of the causes, consequences, prevention and treatment of specific food allergies such as CMA are needed.

In this paper, we report the output from a 2-day meeting with international experts in CMA from various countries in October 2019. The meeting discussed broad topics in allergy prevention and management of CMA including differences in epidemiology, cultural background, eating habits and climate change. This paper will focus on current challenges and future opportunities in CMA, its nutritional management strategies for tapering and reintroducing cow’s milk proteins in the diet, and the potential for prevention which we have combined with recently published developments since the meeting. The conclusions highlight the unmet needs and consequent recommendations for future research for optimal management and prevention of CMA.

## The Ontogeny of CMA and the Allergic March

The immunology of a normal pregnancy involves down-regulating the maternal T-helper-1 lymphocyte (Th1) response to feto-paternal antigens by upregulating T-helper-2 and regulatory T-cell cytokines. These cytokines are present in the amniotic fluid together with any antigens/allergens to which the mother has been exposed. These are swallowed by the fetus, potentially enhancing allergic sensitization through lymphoid accumulations in the small intestine. All neonates consequently have a Th-2 biased immune response which is more entrenched in an atopic maternal environment (22).

A component of balancing the response occurs in the 3rd trimester through allergen aggregated with IgG transported across the placenta which down-regulates sensitization through inhibitory IgG receptors. This being the case, high maternal intake of milk would increase IgG antibodies complexed with milk and when transported across the placenta could reduce the risk of CMA (23). Balancing the Th-1 and Th-2 responses after delivery is affected by a rapidly diversifying ecological balance of organisms (microbiome) on the infant’s skin, in the respiratory and gastrointestinal tracts. A healthy microbiome (Eubiosis) is achieved by 2-3 years of age and is facilitated by pre-biotic oligosaccharides in human breast milk and a diverse healthy diet during weaning (24, 25). Moreover, a mutually beneficial (symbiotic) relationship exists with our microbiome contributing to digestion, absorption, protection against pathogens, health-sustaining immunological and metabolic responses (25).

The allergic march has conventionally presented a sequence commencing with food allergy; in which atopic dermatitis (AD) may be succeeded by asthma and hay fever though allergic rhinitis is more likely to precede asthma. This pre-supposes that the sequence of allergic diseases are manifestations of the same underlying pathophysiology suggesting that one condition leads inexorably to another. However, the gene/environment interactions of each condition are different (26). Allergic sensitization may occur without any clinical manifestations, while AD, asthma and rhinitis can exist separately. Nevertheless, there are some mechanistic explanations for progression through the march for a proportion of subjects. The genetic basis for AD is a defect in skin barrier function of which the best studied is polymorphisms in the gene coding for filaggrin. Defects in this protein lead to increased fluid loss from the epidermis and increased susceptibility to inflammation induced by irritants and micro-organisms, thereby leading to AD (27). The dysfunctional skin barrier is more permeable such that food and inhalant allergens can penetrate through to the dermis where antigen presenting cells can pick up allergen and migrate to regional lymphoid accumulations for sensitization to occur (28). The sequence of the march is, therefore, incorrect with food allergy more likely to be a consequence of the gene defect contributing to AD and more likely to follow rather than precede it.

Given the ubiquity of cow milk in domestic environments, skin contact is inevitable and CMA most likely evolves by this route and certainly is most commonly seen in infants with co-existent AD. Inhalant allergy can also occur by the same route and filaggrin gene polymorphisms are associated with an increased risk of allergic rhinitis and asthma following AD (29). It is therefore, not surprising that allergy to foods, particularly with co-existent AD, is sometimes followed by the development of asthma. While the progression from AD associated with egg allergy to asthma has consistently been demonstrated, there is less evidence concerning milk allergy (28, 30). The allergic march is therefore much more complex, and it may be better to view it as a stop/start dance, such as a tango (31).

## Primary Prevention of CMA

**Figure 1:**
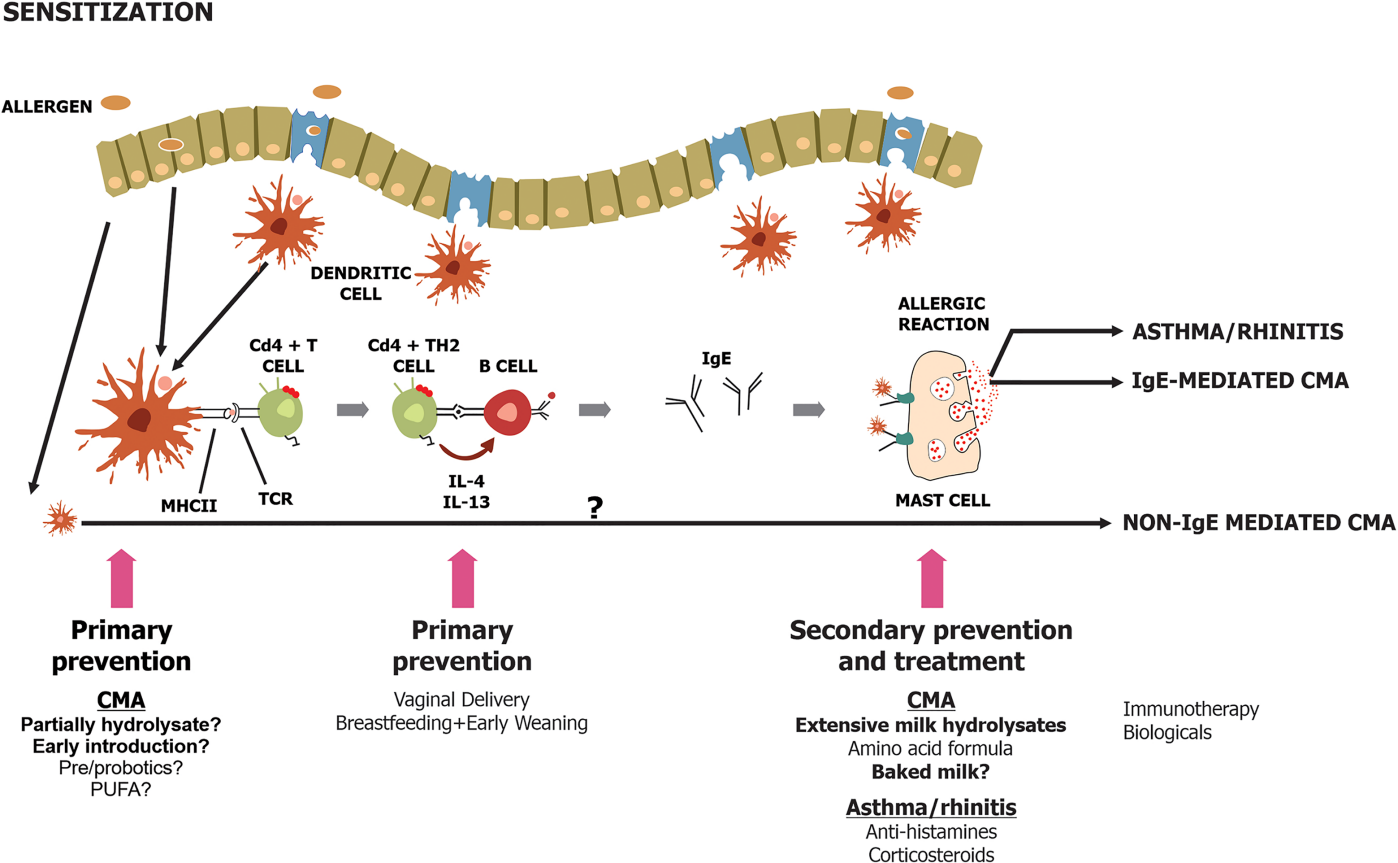
Schematic diagram on primary, secondary and tertiary prevention [adapted from (12)].

Understanding the mechanisms and developing strategies for the prevention of food allergy including CMA is important to curb the increasing incidence of cow’s milk protein allergy (**Figure 1**) (12, 13). Family history of allergy-associated diseases is the most important risk factor for allergy manifestations in the offspring. Primary prevention strategies could be applied in high-risk infants defined as those with a first-degree relative with a history of allergy (32, 33). This section will discuss several potential interventions to prevent the occurrence of CMA in later life.

### Maternal Diet in Pregnancy and Lactation

There is little data available to support the manipulation of the maternal diet during pregnancy or lactation to prevent CMA (34). Kramer and Kakuma conducted a Cochrane Systematic Review which included evidence from five trials, and the overall conclusion was that antigen avoidance during pregnancy and lactation was unlikely to reduce the risk of atopy occurring in their children (35). Several studies showed no reduction in CMA or egg allergy in infants whose mothers were avoiding the respective foods during pregnancy and lactation (36, 37). This is because it is not a linear relationship between exposure levels and allergy risk. It is a bell-shaped curve in which high exposure induces tolerance and very low exposures are not sufficient to trigger a response, while the mid-range level of exposure leads to higher risks of sensitization. Avoidance usually just shifts the bell-shaped curve to the left resulting in no overall change in the population with equal numbers being moved from high dose tolerance to mid-range sensitization and equal numbers from the mid-range to the low dose without sensitization. Trials of maternal high-dose exposures to common food allergens during pregnancy are required. Animal models suggest this approach could confer benefits. Oral ovalbumin in pregnancy can induce tolerance in infant BALBc mice and the protective effect was abrogated by inhibition of placental IgG transfer or infant memory T-cell IFN-γ production (38). A study of 1277 mother-child pairs from the USA unselected pre-birth cohort showed that a high peanut intake in the 1st trimester reduced the risk of developing peanut allergy to 0.53 (0.30-0.94). High milk intake in the 1st trimester reduced the odds ratio for asthma 0.83 (0.69-0.99) and rhinitis 0.85(0.74-0.97) while a high wheat intake in the 2nd trimester reduced the odds ratio for eczema 0.64 (0.46-0.90) (39).

### Breastfeeding

**Figure 2:**
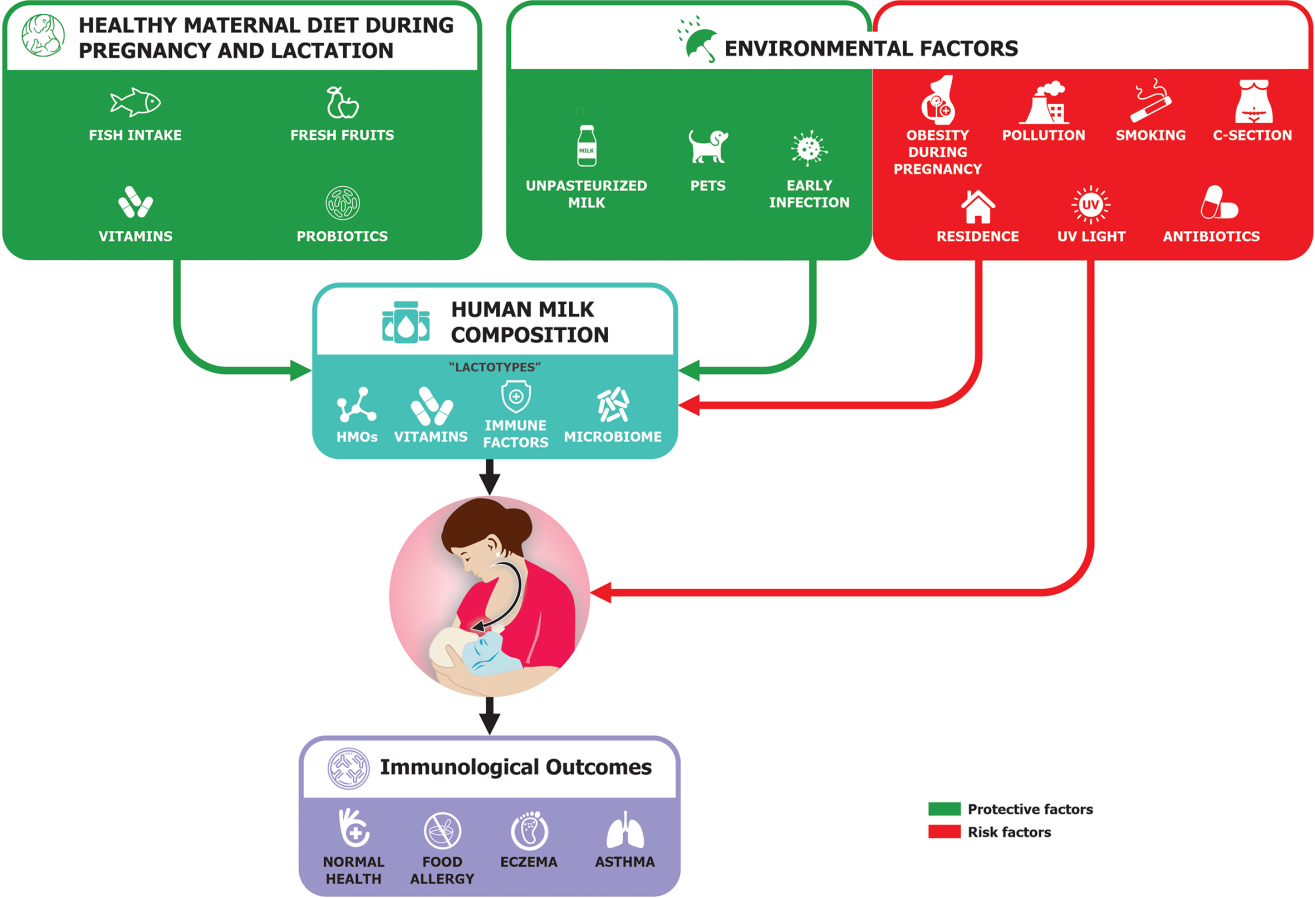
Possible maternal influence on immunological outcomes in child [adapted from Verhasselt (40) and Munblit et al. (41)].

Breastfeeding is an important factor that can lower food allergy development through several mechanisms (**Figure 2**). These include potentially anti-allergic immune properties in the milk, and the possibility that prolonged breastfeeding may delay allergen introduction, but also the presence of antibodies within breast milk may combine with food antigens to induce tolerance (42). In animal models, it has been shown that antigen-immunoglobulin (IgG) immune complexes from sensitized dams were transferred to the newborn through the neonatal Fc receptor resulting in the induction of antigen-specific FoxP3(+) CD25(+) regulatory T cells (43). The transfer of these immune complexes could induce tolerance and support the development of primary prevention of CMA (23, 44).

Recent experimental data reported the role of short-chain fatty acids (SCFA), butyrate in particular, which enhance oral tolerance in the off-spring (45, 46). Likewise, human milk oligosaccharides (HMOs) in breast milk and prebiotics (galactooligosacharides) or a specific HMO (like 2-fucosyllactose) included into infant nutrition can induce the production of SCFAs suggesting that they may play a role in the primary prevention of CMA (47).

Breastmilk also contains various bioactive components such as immune active peptides (cytokines), fatty acids, HMOs, microbial contents and micronutrients which have been proven to modulate the immune system (41). One of the longest cohort studies, the PROBIT trial, has recently reported that breastfed infants had a lower risk of developing flexural dermatitis at 18 years of age. However, there was no effect on lung function, the incidence of CMA and asthma (48).

### (Partially) Hydrolyzed Formulae for Non-Exclusively Breastfed Infants

Partially hydrolyzed formulae (pHF) generally contain peptides with molecular weights around < 5,000 Da (49). The partial hydrolysis removes part of the sensitizing epitopes which reduces the allergenicity of the proteins and their ability to induce sensitization, while retaining sufficient size of peptides to stimulate the induction of oral tolerance (50, 51).

The role of partially hydrolyzed formulae (pHF) for primary prevention of CMA has been long debated (52). The longest, largest, longitudinal studies on pHF, the German Infant Nutritional Intervention Study (GINI study) which started in early 2000 showed that whey pHF was significantly associated with reduction of AD manifestations up to the age of 15 years old (53–57) and reduced the risk for asthma and other respiratory symptoms (58). However, a more recent study with pHF enriched with pre-biotic oligosaccharides did not show any preventive effect (59).

Furthermore, there is no consensus with regards to early exposure to intact CMP in the first few weeks of life and the risk of CMA in later life. A large observational cohort study showed that delayed introduction of cow’s milk products in the infants’ diet was associated with an increased risk of developing atopy at 2 years of age, especially AD (60). A prospective study assessing the risk factors for CMA found early (within 14 days of life) introduction of CMP formulae presented lower rates of IgE-mediated CMA compared to those with late consumption at 105-194 days of life [0.05% versus 1.75% respectively (p < 0.001)] (61). A recent study showed that early continuous exposure to CMP may reduce the risk of CMA; while an introduction to CMP formulae during the first 3 days of life followed by complete CMP avoidance until the child is weaned may have the opposite effect (62). The difference in outcome may be influenced by the family history.

From a health economic perspective, a cohort using a Markov model simulating the AD incidence and burden from birth to age 6 years in a target population fed with whey pHF vs CMF, suggested that feeding a high-risk infant whey pHF resulted in a 14% reduction in AD risk (95% CI, 3%–23%) and a per-child net saving of MYR 1,113 (US $352) (95% CI, MYR 317–1,884) favoring whey pHF (63).

The European Academy of Allergy and Clinical Immunology (EACCI) recommends exclusive breastfeeding in the first few weeks of life. If needed, a hydrolyzed milk formulae could be recommended under medical guidance to reduce the risk of AD depending on the clinical, economic and cultural factors (64). This recommendation follows the latest Cochrane review in 2018 on pHF and allergy prevention which states that there is no evidence to support feeding with pHF for the prevention of allergic diseases (65). The change in recommendation could be most probably driven by the negative results reported in the recent studies (59).

### Timing of Weaning Food Introduction

The concept of oral tolerance is well-documented from previous work showing how early and regular oral exposure induces clinical tolerance and altered immunological responses to food allergens (32, 66). Further research in humans has also shown that early ingestion of food allergens can lead to oral tolerance while skin contact before tolerance has been achieved, particularly in the presence of inflammation, epidermal barrier defects and AD, leads to sensitization (32). The evidence supporting the role of early introduction of potential allergens in the development of oral tolerance to prevent food allergy is mounting. Although there are still questions as to the timing and also which allergens can be introduced safely and with effect, a shift from recommending avoidance of common food allergens to early consumption strategies to prevent the development of food allergy is occurring. The LEAP study and EAT study reported the benefit of early complementary feeding with egg and peanut allergy, but an equivocal effect for milk. It was not clear whether early introduction of milk and other allergens was useful to reduce the risk of allergy due to difficulty in complying with the regimen in the study (67, 68). Furthermore, the EAT study also reported that the rates of non-IgE symptoms at any time point were equivalent between groups suggesting that the introduction of the study foods was associated with the reporting of these symptoms regardless of when they were introduced. Specifically, 11.9% of participants in the early introduction group reported non-IgE type symptoms to one or more of the early introduction weaning (69) foods at any point (4-12 months) compared to 9.6% of the standard introduction group (p = 0.20). However, this effect on non-IgE mediated allergy may indicate that there was not enough exposure to alter immune responses or were due to other causes.

AAP in 2019 recommended introducing peanut between 4 – 12 months in countries with a high prevalence of peanut allergy and well-cooked egg between 6 – 8 months (70). This is further emphasized in the recent EAACI guidelines which stated that in countries where egg allergy is an issue, providing well-cooked egg twice a week between the age of 4-6 months can be recommended (64). There is no reason for delayed introduction >12 months nor for an early introduction <4 months. Experts recommend consideration of local data, dietary practices and family preferences and assessment of the child’s and family atopic status when introducing early foods during weaning to reduce the incidence of food allergy.

In addition to weaning foods, timely introduction to intact cow’s milk in non-high risk infants perhaps around 4-6 months is likely to be of value (69, 71)

### Environmental Factors

Interestingly, epidemiological evidence points to the role of unprocessed farm milk as protective in the development of asthma and allergy. In several large epidemiological studies, the consumption of unprocessed farm milk and exposure to the farming environment were associated with a lower incidence of asthma and allergy (72, 73). To date two studies have tested the exposure of cow’s milk allergic children after weaning period to raw milk as compared to regular shop milk, and in both these studies raw milk led to fewer medical complaints related to asthma and allergies (74, 75). Collectively these findings suggest that unprocessed milk contains factors that can prevent the development of clinical allergy due to the influence of cow milk’s microbiome and/or other immune active constituents.

### Topical Emollients

As a compromised skin barrier and the presence of dermatitis predisposes to percutaneous sensitization risk to food antigens, there is the possibility that good skin care aiming at active AD control may decrease allergen sensitization and the subsequent allergy march (76). Both the ecological perspective (77, 78) and basic cutaneous biology (79, 80) have suggested that epidermal barrier dysfunction is a key initiator to the development of AD. The use of emollients in reducing eczema is still inconclusive. Some studies show a reduction in eczema development with no reduction of food sensitization. However, recent evidence reports that the use of emollients from birth may not be protective against the onset of AD in infants and young children (81).

### LCPUFAs

Maternal diets high in omega-3 long-chain polyunsaturated fatty acids (LCPUFA) are thought to have a protective effect against the development of allergies in the newborn (82). Supplementation with docosahexaenoic acid and eicosapentaenoic acid during pregnancy has been shown to increase LCPUFA concentrations in breast milk (83). A large randomized clinical trial of maternal fish oil supplementation (500 mg of docosahexaenoic acid and 150 mg of eicosapentaenoic acid) during pregnancy demonstrated a significant decrease in cord blood concentrations of Th-2 cytokines (IL-4 and IL-13) as well as increased levels of oral tolerance-inducing TGF-beta (84). However, this study showed no effect on clinical outcomes. Palmer et al. assessed the effect of high-dose fish oil supplementation (800 mg) in high-risk infants (with a positive family history of atopy) on infantile eczema and food sensitization at 12 months of age. Those receiving the fish oil supplementation had significantly lower rates of atopic eczema and egg sensitization. High intake of omega 3 LCPUFA *via* fish oil supplementation or high intake of oily fish during pregnancy and after birth seems to reduce the risk of AD and food sensitization to common allergens and asthma but not specifically to CMP (85, 86). This suggests the influence of dose and other risk factors can impact specific allergic outcomes and not just a single interventional factor.

### Modulation of the Gut Microbiome With Prebiotics and Probiotics

The latest Cochrane review in 2013 reported a potential benefit of prebiotics during infancy in the prevention of AD, but no conclusive evidence was found regarding the prevention of other allergic diseases or food allergies (87).

Human Milk Oligosaccharides (HMO) are complex, nondigestible oligosaccharides with prebiotic properties in breast milk which provide a specialized substrate for Bifidobacteria and enhance an ecological balance associated with eubiosis directly or indirectly *via* SCFA production (88). Over the past decade, several manufactured prebiotics have been added to infant formulas, including plant-based long-chain fructo-oligosaccharides (FOS) and short chain galacto-oligosaccharides (GOS). GOS and FOS have been shown to increase counts of fecal Bifidobacteria in formula-fed infants (89, 90). A randomized trial of a GOS/FOS with 9:1 ratio supplemented pHF investigated the effect on AD in formula-fed infants during the first 6 months of life. In that study, the GOS/FOS group had significantly lower rates of AD compared to the placebo group, although severity was similar for both treatment arms (91). A more recent European multi-center randomized controlled trial assessed the effect of neutral oligosaccharides (GOS/FOS) and pectin-derived acidic oligosaccharides in a standard infant formulae in healthy, low-risk infants from 8 weeks to 12 months (92). These prebiotics reduced the incidence of AD by 44% at 12 months with no effect on disease severity. However in a more recent study, there was no effect of GOS/FOS on AD incidence, although immunological differences and effects on the gut microbiome resembling those receiving human milk was noted in those receiving GOS/FOS supplemented formula compared with standard formula were noted (59). The dynamics of the evolving microbiome in these infants were different in those who developed AD suggesting a further influence of GOS/FOS on the microbiome in the development of oral tolerance as compared to intact protein milk formula (59, 93).

HMOs in breast milk provide the substrate for specific microbes and significantly influence early microbial gut colonization (94, 95). In preclinical studies, HMO has been shown to attenuate allergic responses in cow’s milk-sensitized mice (96). The role of HMO in the prevention and treatment of CMA represents a promising area for future research (96–98).

An inverse association between microbial exposure in early life and allergy was suggested by the hygiene hypothesis (99). Since then, there has been increasing interest in diet supplementation with live bacterial cultures (Lactobacillus rhamnosus GG, Lactobacillus acidophilus, Lactobacillus reuteri, and mixed cultures). They have protective gastrointestinal effects modulating immune responses and contribute to the regulation of theTh1/Th2 ratio and stimulation of the regulatory cytokine IL-10. A meta-analysis of randomized controlled clinical trials investigating the use of probiotics in infants for primary prevention of allergies found a small reduction in AD in infants, but insufficient evidence for a general recommendation of probiotic supplementation for prevention of allergic disease or food hypersensitivity (100).

The World Allergy Organization and Mc Master University Guidelines for Allergic Disease Prevention (GLAD-P) panel of experts indicated a lack of robust evidence confirming the effectiveness of strain-specific probiotics in a group of pregnant or breastfeeding women, or infants at a high risk of atopy on prevention of food allergy (101). However, its effect on reducing the risk of AD has been reported in a meta-analysis (100) and in infants (102). No studies have reported the effect of supplementing strain-specific probiotics during pregnancy or early life in primary prevention of CMA (103, 104). However, the effect of mixed strains of probiotics, or synbiotics (a mixture of pre- and pro-biotics) to achieve greater microbiota diversity has been shown to reduce the risk of necrotizing enterocolitis in premature infants. The same might be true for the primary prevention of food allergy and is worthy of further investigation.

## Secondary Prevention and Management of CMA

Preventing disease progression from mild or moderate symptoms to severe symptoms or another allergy phenotype in children with CMA is considered as secondary prevention and management of CMA (33).

### Extensively Hydrolyzed Formulae

Whey- or casein-based eHF are considered the first-line management of formula-fed infants with CMA (13, 17, 49, 105). These formulas contain short cow’s milk peptides that are produced *via* enzymatic breakdown and ultrafiltration of intact cow’s milk proteins. There are significant differences in the molecular weights and profiles of peptides in eHF (106, 107). A task force of the European Academy of Allergy and Clinical Immunology (EAACI) has therefore called for stricter standards for the definition of eHF marketed in Europe, including preclinical testing, quality assurance, and labelling requirements (104). In 2016, DRACMA updated their recommendation to include the provision of rice hydrolysate in the first-line management in countries where this formula is available (108).

### Amino Acid-Based Formulae (AAF)

AAF is a synthetic, nutritionally complete, cow’s milk antigen-free formulae containing free amino acids, which is used in the treatment of infants with severe CMA. Its cost-effectiveness in clinical practice is dependent on the healthcare system and cost of formula in a particular country (109, 110). AAF is therefore not a first-line treatment but recommended for infants who have failed treatment with eHF (17), as well as infants with very severe symptoms such as cow’s milk anaphylaxis (111) or multiple food intolerances (112). As tolerance development is thought to be an antigen-driven process, the use of AAF is unlikely to promote tolerance development (113) although a recent study showed that supplementing AAF with synbiotics improved the gut microbiota in non-IgE mediated allergic infants (114). The use of AAF for more than 6 months in children was recently reported to have the potential adverse effect of causing hypophosphatemic bone disease especially among those with complex GI conditions (115–119). These studies also reported restoration of calcium and phosphorus homeostasis following a formula change to eHF. In addition, data from healthy volunteers in an acute setting did not indicate differences in calcium and phosphorus bioavailability from various types of AAF even if combined with an acid-suppressant medication (120).

### Partially Hydrolyzed Formulae (pHF)

It may be prudent to consider the role of pHF in the management of CMA under certain conditions, such as mild cases of CMA or weaning from eHF before introducing intact cow’s milk protein. Given the highly controlled manufacturing process in the production of pHF, they provide potentially a safer alternative compared to using either baked milk, or small doses of heated milk (3 ml), both of which were shown to be successful in enhancing tolerance to the unheated CMP, as compared to allergen avoidance regimens (121, 122). Around 72% (out of 55 tested subjects) of non-IgE allergic children were able to tolerate pHF in an open food challenge without any adverse events (123). In another recent study, Inuo et al. also demonstrated an increased tolerance in allergic children towards higher quantities of pHF compared to intact CM and found no significant differences between the pHF and eHF in the rate of tolerance or induction of allergic symptoms (51). It has been hypothesized that partially hydrolyzed proteins (whey- or casein-derived peptides of various molecular weights) will result in better oral tolerance induction in a setting of immature gastrointestinal and immune systems, compared to intact CMP.

### Modulation of Gut Microbiome With Prebiotics and Probiotics

In addition to the previously mentioned FOS and GOS as prebiotics, a recent publication reported the hypoallergenic effect of eHF supplemented with two human milk oligosaccharides (HMO), namely 2’fucosyl-lactose (2’FL) and lacto-N-neotetraose (LNnT) as compared to eHF only among children with CMA. The study reported that the eHF supplemented with HMOs is safely tolerated by infants with CMA (124).

Provision of strain specific probiotics, especially Lactobacillus GG (LGG) in management of CMA and induction of oral tolerance have been reported in several studies (125). eHF with LGG has been reported to have the absolute risk difference for the occurrence of at least 1 allergic manifestation over 36 months of –0.23 (95% CI –0.36 to –0.10; p < 0.001) and the absolute risk difference for the acquisition of cow’s milk tolerance was 0.20 (95% CI 0.05–0.35; p < 0.01) at 12 months, 0.24 (95% CI 0.08–0.41; p < 0.01) at 24 months, and 0.27 (95% CI 0.11–0.43; p < 0.001) at 36 months as compared to eHF without LGG. The addition of LGG has also significantly increased fecal butyrate levels which have been reported to potentially facilitate oral tolerance induction (45, 126).

## Step-Down Approach to Build Early Tolerance in Re-Introducing Intact Cow’s Milk Protein

By providing the pHF in between AAF or eHF and full cow’s milk as part of a step-down approach in the management of CMA, it is postulated that the children might achieve tolerance to CMP sooner (49). There are differences between eHFs in the degree of hydrolysis, which affects the distribution of peptide size (127).

Common casein-based eHF generally have a high degree of hydrolysis (eg 37%) and 80% of peptide less than 500Da, 10% of peptides between 500-1000 Da and 10% of peptides larger than 1kDA (127). On the other hand, the majority of eHF whey-based formulas have a lower degree of hydrolysis (e.g. 22%) resulting in a smaller percentage of peptides between 500 Da-1kDA and remaining 40%>1 kDA (127). We hypothesize that having two different eHFs with different degrees of hydrolysis could be useful as an intermediate step between extensive hydrolyzed, in general, and partially hydrolyzed protein. For the ease of differentiation, we will refer to the hydrolysates with has a lower degrees of hydrolysis as Intermediate Hydrolyzed Formula (iHF).

In allergen immunotherapy, tolerance to inhalant allergens is induced by injecting allergic individuals with low doses of allergens, to enhance non-IgE antibodies (most notably IgG4) and enhance the regulatory T-cell generation of TGF-b and IL-10 (128). By analogy, hydrolysates that do not retain IgE-binding epitopes (>30 aa) but retain CD4+ T cell epitopes (12-18 aa) may modulate T cell responses to the allergen in the absence of inducing IgE-mediated allergic responses (129).

In an *in-vitro* mode, different lengths of peptides have been shown to influence the local and systemic immune response especially in strengthening the gut epithelial barrier *via* increasing the regulatory cytokines (ie IL-10) and decreasing pro-inflammatory markers such as cyclo-oxygenase2 (COX-2) and IL-8. Peptides also have been shown to modulate immune responses *via* direct interaction with Toll-like receptors (TLR) which are responsible for interaction between pathogen and the gut mucosa but are also suggested to have an immune-modulatory role in allergic inflammation (130). The TLR activating capacity of whey hydrolysates decreased with the increasing level of hydrolysis (131).

**Figure 3:**
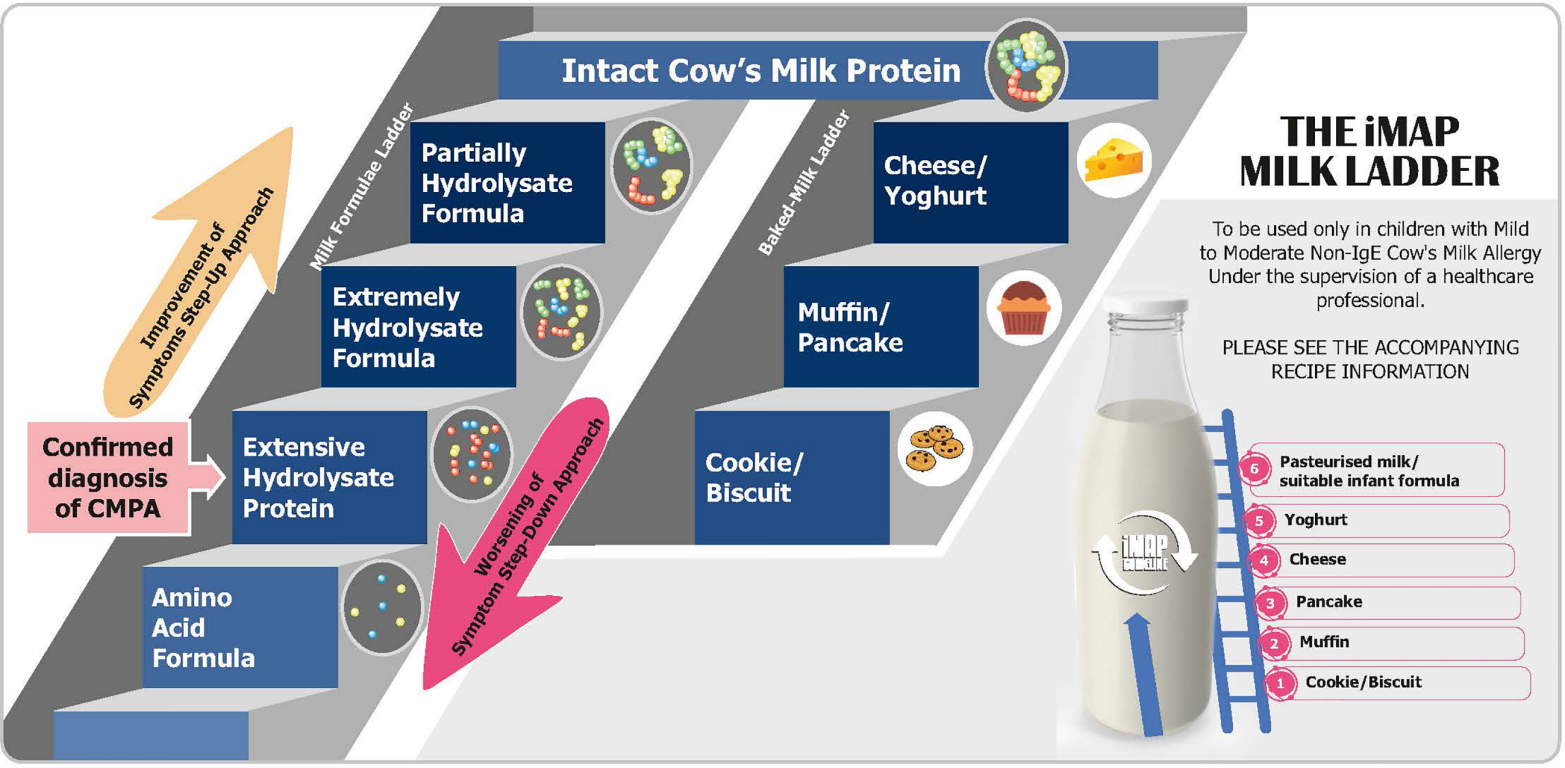
Milk formulae and baked-milk ladder step-down and step-up to support early tolerance in management of CMPA.

The step-down approach for children with CMA using a hydrolysate milk ladder incorporating pHF as a bridge between extensively (eHF) and the intact CM formulae is currently implemented in Greece under careful monitoring. It is suggested that one more step is added from casein to whey based eHF (iHF) and then to pHF formula, after stringent evaluation of the individual’s clinical history and skin prick test’ wheal size to each hydrolysate formulae (**Figure 3**). A clinically monitored oral challenge to the respective formulae is conducted before allowing consumption at home.

There are several so-called milk ladders from baked to non-baked food that is currently recommended in Europe to slowly reintroduce cows’ milk proteins into an infants’ diet when it is assumed that the allergy is resolving. An example commences with a malted milk biscuit followed if tolerated successively by cheesy breadsticks, custard, a teaspoon of yoghurt, cheese, and finally pasteurized milk (132). The issue with milk ladders is that the levels of CMP in each step are uncharacterized and likely variable dependent on the degree of heating and/or processing and are based on observational rather than controlled studies.

Ideally, formulas with various degrees of hydrolysis are needed in a hydrolysate milk ladder. In that way, the introduction of peptide to protein fragments can be more controlled and will eliminate the presence of several biases such as the presence of other potential allergens. However, considering the cost of such formulas and the ease of baked-milk food during the weaning period, this step-wise proposal using various degree of hydrolysate needs to be proven in a clinical setting to be cost/effective as compared to current milk ladders before recommending it for routine practice. Before robust clinical evidence is available, this step-wise proposal should be implemented in a clinical setting with stringent medical guidance; and in selected cases such as among children with confirmed Ig-E mediated CMA.

Around 50% of infants receiving eHF will have developed tolerance by the age of 12 months (133). With the above-mentioned step-down proposal, it is hypothesized that there could be a possibility for an increase of more than 50% of children who would gain tolerance at 12 months or shorter duration than 12 months for developing tolerance. These outcomes will have a significant impact on reducing the health and economic burden of the affected families.

Tolerance will be determined by conducting oral food challenges as the preferred diagnostic standard. In principle, food challenges include seven doses for both the active and the placebo arm of the challenge (in the case of DBPCFC) as suggested by previous recommendations and the EuroPrevall and iFAAM projects (4, 134).

Other mammalian-based formulas (e.g. sheep, goat) will not be tolerated by the majority of subjects with CMA. Soy-based formulas are not recommended for use under the age of 6 months of age in many European and American Allergy guidelines, and other plant-based milk *(e.g. rice, almond, oat)* could be nutritionally inadequate (135).

## Unmet Need and Future Research

Several studies on migrating families revealed a big discrepancy in the prevalence of food allergy if there is a mismatch between the pre-conception, antenatal and postnatal environment. For example, if the mother conceives and gives birth in a new country, the baby has a higher risk of food allergy and AD compared to babies who were born in the country of maternal origin (136, 137). This amply illustrates that environmental factors rather than genetics had contributed to the increasing prevalence of food allergy. As such, there is therefore a greater chance that environmental modification will favorably impact on allergy risks in the future. Based on the evidence, we believe that a multi-faceted approach to prevention should be submitted to controlled clinical trials. These should be employed during pre-conception and pregnancy with an optimized diverse diet and no food allergen avoidance, no smoking, antibiotic avoidance if possible, and avoidance of a caesarean section delivery. If either of the latter is not possible then administration of pre/pro/synbiotics to the mother during pregnancy is indicated. Post-natally the ideal is breast feeding but if not possible, whole milk formula supplemented with pre-and probiotics. Rapid weaning onto the common allergenic foods beyond 4 months of age, with a particular focus on peanut and egg is proposed for both breast and bottle-fed infants. Preferably, weaning should proceed while breast feeding is continued.

Intervention during pregnancy or pre-pregnancy remains the best option to prevent CMA in the offspring as there is evidence that the high levels of specific IgG to a particular allergen at birth reduces the risk of allergy. For example, a study of ryegrass immunotherapy during pregnancy showed high levels of a specific IgG to ryegrass and reduced risk of ryegrass allergy compared to those born to mothers who are allergic and didn’t receive immunotherapy during pregnancy (138).

For postnatal intervention, if the mother is a high milk drinker during pregnancy, then it would seem rational to give milk proteins postnatally early because having IgG milk protein complex transfer across the placenta should be matched with early tolerance induction before sensitization can occur *via* non-GI tract exposure. This requires controlled trials to establish if it is effective. On the other hand, if the mother has a very low milk intake, it may not be appropriate to suddenly give milk proteins post-natally. High milk intake during pregnancy is associated with a reduced risk of asthma and allergic rhinitis at 8 years of age (39). There could be some correlation between the low maternal milk intake and the high incidence of CMA in offspring. Therefore, there is likely to be no one-size-fits-all approach to prevent CMA, and clinical judgment may be based on maternal milk exposure during pregnancy. All the suggested strategies above require controlled clinical trials before being employed as routine practice.

There is a need to conduct a controlled, prospective follow-up study in babies with AD and appropriately confirm CMA to evaluate the impact of early introduction of a milk ladder using progressively less hydrolyzed milk formulae on tolerance development to CM and the subsequent development of other allergic disorders later in childhood compared to the current standard of waiting for 6–12 months for re-challenge (49, 69, 71). If this approach is successful in inducing the early development of tolerance to cow’s milk protein, it could have considerable health economic benefits.

## Conclusion

Increased incidence of food allergy, including CMA, among infants and young children, has been observed over the last decade (3, 139). Its primary prevention should include having food diversity during pregnancy to ensure the transfer of IgG-allergen complexes prenatally (and by breast milk postnatally) to provide early tolerizing exposure to the fetus. In addition, continued exposure to milk among milk-drinking breastfeeding mothers postnatally should be continued. For non-breastfed infants at risk to developing CMA, partially hydrolyzed formulae with proven efficacy may provide health-economic benefit among high-risk infants with a family history of allergy (140, 141) but further trials are required.

Secondary prevention in the management of CMA should focus on the use of eHF mainly due to its immune-modulating capabilities to induce tolerance as compared to AAF. We hypothesize that the introduction of hydrolysates with various degrees of hydrolysis may help in building faster oral tolerance and thus can be considered in a milk formula ladder, in addition to clinically proven pre- and probiotics. All of these proposals require further controlled trials before they can be recommended for routine practice.

## Author Contributions

All authors were involved in setting the concept and objective for the review. BZ-O contributed the first draft which was then further developed by LM, JN, UK, and JW based on the meeting report and latest publications. All authors participated in the development of the final manuscript by providing comments and suggestions. All authors contributed to the article and approved the submitted version.

## Funding

FrieslandCampina provided an educational grant to develop the 2 figures in the manuscripts to SovereignHealth, Singapore and funded the 2-day meeting. None of the authors received funding from FrieslandCampina to write the manuscript.

## Conflict of Interest

UK, JN, and LM are employees of FrieslandCampina.

The remaining authors declare that the research was conducted in the absence of any commercial or financial relationships that could be construed as a potential conflict of interest.
